# Link between MnSOD Ala16Val (rs4880) polymorphism and asthma risk is insignificant from sequential meta-analysis

**DOI:** 10.6026/97320630016789

**Published:** 2020-11-30

**Authors:** Hazza A Alhobeira, Raju K Mandal, Saif Khan, Sajad A Dar, Harishankar Mahto, Mohd Saeed, Mohd Wahid, Mohtashim Lohani, Mahvish Khan, Shafiul Haque

**Affiliations:** 1 Department of Restorative Dentistry, College of Dentistry, University of Ha'il, Ha'il-2440, Saudi Arabia; 2 Research and Scientific Studies Unit, College of Nursing & Allied Health Sciences, Jazan University, Jazan-45142, Saudi Arabia; 3 Department of Basic Dental and Medical Sciences, College of Dentistry, University of Ha'il, Ha'il-2440, Saudi Arabia; 4 Center for Life Sciences, Central University of Jharkhand, Ranchi-835205, Jharkhand, India; 5 Department of Emergency Medical Services, College Applied Medical Sciences, Jazan University, Jazan - 45142, Saudi Arabia; 6 Department of Biology, College of Science, University of Ha'il, Ha'il-2440, Saudi Arabia

**Keywords:** Asthma, genetic model, meta-analysis, MnSOD, polymorphism, susceptibility

## Abstract

The mitochondrial manganese superoxide dismutase (MnSOD) enzyme protects lungs against oxidative stress by neutralizing the free radical superoxide produced in the respiratory function. This has relevance to asthma. Therefore, it is of interest to describe the potential effect of MnSOD Ala16Val genetic polymorphism to asthma risk. Known data in this context is inconclusive in nature. The possible link between MnSOD Ala16Val polymorphism and asthma is explored using sequence meta-analysis. Data from the pooled analysis of MnSOD Ala16Val polymorphism using five genetic models i.e., allelic (Val vs. Ala: p=0.846; OR=1.033, 95% CI=0.742 to 1.440) is discussed. Homozygous (Val Val vs. Ala Ala: p=0.517; OR=1.307, 95% CI=0.582 to 2.932) and heterozygous (Val Ala vs. Ala Ala: p=0.307; OR=1.138, 95% CI=0.888 to 1.459) data using the described models are documented. Data from the dominant model (Val Val + Val Ala vs. Ala Ala: p=0.301; OR=1.289, 95% CI=0.797 to 2.085) and the recessive model (Val Val vs. Val Ala + Ala Ala: p=0.761; OR=0.924, 95% CI=0.555 to 1.538) analyses for several ethnic subgroups in this context is reported.

## Background

Asthma is a complex disease, chronic in nature and is highly prevalent across countries, which affect almost 334 million people in all age group [1]. It causes significant mortality and morbidity and reduces quality of life. Despite being major advances in the treatment of asthma and the development of several guidelines at government level in different countries over the past few decades, the world is continuously noticing many people dying of the mentioned disease. The World Health Organization estimate shows around 383,000 asthma deaths in 2015. The rising trends show that it can increase to 400 million, by 2025 (http://www.who.int/ mediacentre/factsheets/fs307/en/). Despite the fact that asthma etiology has remained unclear for long, the studies have shown it as a genetic disorder of complexity, which is characterized by inflammation of the airway and reversible airflow obstruction [2]. Previous studies have demonstrated that the interaction between genetics and the environment is one of the main factors responsible for asthma pathogenesis [3]. As, the complexity and severity of this disease continue to increase and bearing the greatest global challenges and medical threatens, it is important to identify the genetic risk factors that adds to the considerable burden of the disease which further warrant potential screening and therapeutic strategies for control and prevention. The use of genome-wide association studies (GWASs) tool is becoming indispensable for the identification of common variants that are associated with asthma. The GWAS have successfully reported single nucleotide polymorphisms (SNPs) in genes related to asthma susceptibility playing an important role in etiology of asthma [4].

Lungs are more exposed to oxidative damages because of continuous higher oxygen tension prevalence. The body has an active defense antioxidant system to protect the lung tissues against oxidative injuries caused by various genes responsible for oxidation. The gene encoding the enzyme mitochondrial manganese SOD (MnSOD) is located on the long arm (6q25) of chromosome 6. This arm contains five exons and four introns with each subunit consisting of 196 amino acids [5]. It is one of the most important intra mitochondrial enzymes destroying the superoxide anions produced during mitochondrial energy metabolism, and converts O2- into hydrogen peroxide (H2O2) [6]. It also plays a significant role in controlling dioxygen toxicity in mitochondria, which has an extreme oxidative load [7]. The SNPs of MnSOD antioxidant gene has the capacity to change enzyme structure, substrate specificity or activity, leading to modifications in variability among individuals in oxidative stress defense capability.

The MnSOD encoding gene contains SNP, which has genetic variants comprising of a structural mutation wherein cytosine (C) changes to thiamine (T) in the exon 2. The above stated substitution further affects codon number 16, mutating alanine (GCT) to valine (GTT) changes at the 9th position (Ala16Val, rs4880). This polymorphism alters the helix structure and variant allele formed as result of this produces a β-sheet secondary structure in place of the expected α-helix, which can lead to increase in the production of H2O2 and contribute to the accumulation of ROS [8]. Since, MnSOD gene involved in regulation of the antioxidant defense system, most of the researchers have performed the genetic association study between Ala16Val polymorphism and asthma development to demonstrate that this polymorphism could be a main host susceptibility factor in asthma occurrence [9-13]. However, the results published in various articles are inconsistent and inconclusive. The reason for inconsistency in the results across different case-control studies could be because of individual studies of small sample size with less statistical power. Nowadays, meta-analysis has become an important statistical tool that can be used for pooling the results from large number of individual studies in order to increase the sample size and produce an accurate and a reliable conclusion [14]. Therefore, it is of interest to describe the potential effect of MnSOD Ala16Val genetic polymorphism to asthma risk using sequential meta-analysis.

## Materials and Methods:

### Identification of germane studies:

A web-based systematic search was performed for the studies having association between MnSOD Ala16Val gene polymorphism and asthma. The online databases searched for the current study were PubMed (Medline), Google Scholar, and EMBASE. The last search was updated on 28th February 2019 to retrieve the most compatible and peer reviewed research articles. The combination of the words used for the search was: 'mitochondrial manganese SOD OR MnSOD AND Asthma OR Asthmatic susceptibility OR risk in combination with single nucleotide polymorphism (SNP) OR polymorphism OR genetic variant OR mutation. The articles retrieved were assessed by their titles and abstracts. The ones matching the established eligibility criteria were taken up for this meta-analysis. We also did some manual search of the references listed in the retrieved articles for other eligible articles.

### Inclusion and exclusion criteria:

**Figure 1:**
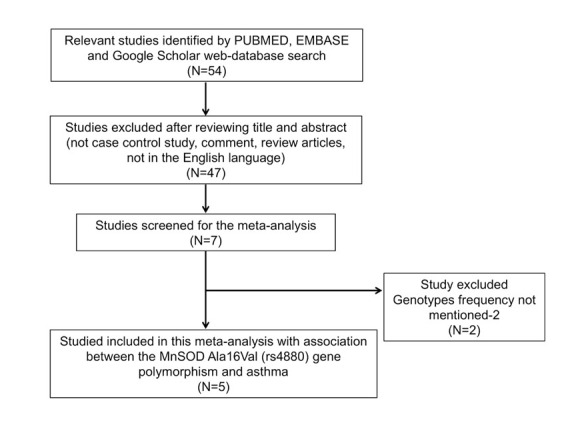
PRISMA 2009 flow-diagram showing the identification and selection process (inclusion/exclusion) of the eligible studies for the present meta-analysis

The criteria used for the inclusion of pertinent research articles in the current meta-analysis study were: (a) it should have evaluated the association between MnSOD Ala16Val gene polymorphism and asthma risk, (b) should have used a case-control study design, (c) should have done the recruitment of confirmed asthma cases and healthy controls, (d) should have the availability of genotype frequency in both case and control studies to count the odds ratio (OR) and must have 95% confidence intervals, (e) the publication of the studies should be in English language. In the same way, there were some preset criteria applied for the exclusion of studies as well. The key basis for the exclusion of the studies were: (i) studies not designed as case-controls, (ii) reviews, abstracts only or overlapping studies, (iii) studies with no reported frequencies for genotypes. The schematic flow diagram depicting the selection of eligible studies, following the established criteria for inclusion and exclusion, for this meta-analysis is shown in Figure 1 (PRISMA 2009 Flow Diagram).

### Literature search strategy:

Two investigators (FLA & RKM) independently evaluated all the titles and abstracts available from the retrieved publications using the selected online databases search in a chronological manner. The full-texts of all the publications, deemed potentially qualified, were retrieved. In order to assess the eligibility for the inclusion of the study, one investigator (RKM) thoroughly appraised all the full-text articles. Following this step, the second investigator (FLA, SK, ML) repeated the same evaluation process independently by randomly selecting 10% of the full-text articles. The two investigators were in full agreement regarding the selection criteria and study exclusion. Subsequently, for identification of the final set of the eligible articles, a third investigator (SK) extrapolated the relevant data from all the studies. To crosscheck the above step, a fourth investigator (MK and ML) independently reappraised the collected information from all the included studies. Any discrepancy associated with the study selection was resolved by a comprehensive discussion with fifth investigator (SH and SAA), who contributed as an arbitrator.

### Screening of the studies:

The electronic searches performed on selected web-databases fetched out fifty-four research articles in the initial stage. But, after many rounds of screening by applying above stated pre-set selection (inclusion/exclusion) criteria only five studies were found eligible to be included here in this analysis. The selected five studies dealt with MnSOD Ala16Val gene polymorphism and occurrence of asthma risk. Two types of studies were straightway excluded which involves MnSOD polymorphism to predict survival in asthma patients and those considering MnSOD variants as indicators for response to therapy. Similarly, studies indicating the levels of MnSOD mRNA or protein expression and other relevant review articles were also disqualified. This study includes only case- control studies reporting the frequency of all the three genotypes. Furthermore, in addition to the database search, the reference lists of the available eligible retrieved articles were also scrutinized for other potential articles, but no pertinent studies fitting the above criteria were found.

### Quality assessment of the studies using Newcastle-Ottawa Scale (NOS) criteria:

Two investigators (SAD, ML & SK), independently, evaluated the quality of the selected studies for this meta-analysis by using the preset NOS criteria [15]. The preset NOS criteria mostly comprised of three aspects: (i) selection of the subjects: 0-4 points; (ii) comparability among subjects: 0-2 points; (ii) clinical outcomes of the studies: 0-3 points. The selected studies acquiring 5 points/stars or more were considered as of moderate to high quality [16]. Any discord between the two investigators was resolved by systematic discussion or expert consultation, if necessary, with the arbitrator (SH and SAA).

### Data extraction:

The methodological data extraction was performed independently in duplicate. It was done independently by FLA,RKM,MK following a standard validated protocol. Using the strict preset selection criteria in standard data collection form ratified the accuracy of abstracted data. The major characteristic abstracted was name of the first author, year of publication, the country of origin, the number of cases and controls, type of study, genotype frequencies, and association observed for cases and controls. Any disagreement or discrepancies in the items abstracted were fully resolved to the point of agreement following many rounds of discussions involving the adjudicator (SH and SAA).

### Publication bias diagnosis & Heterogeneity evaluation:

The publication bias present in the included studies was checked by funnel plot asymmetry and Egger's regression statistics. A p-value of less than 0.05 was fixed for considering the significant publication bias. Additionally, all included MnSOD gene polymorphism related studies were checked for heterogeneity using Q-test and I2 statistics.

### Statistical analysis:

The association strength between MnSOD Ala16Val gene polymorphism and the risk of developing asthma was evaluated precisely using OR and 95% CI. The pooled ORs were calculated for allele contrast, log-additive, dominant, and recessive genetic models [17]. Heterogeneity assumption between the studies across the eligible comparison was done by the chi-square-based Q-test [18]. Heterogeneity was considered significant, if the p value was found to be less than 0.05. Both the fixed effect and random effect models were applied for pooling of the results if p-value was less than 0.05 [18-19]. In addition to the above-discussed methods for statistical analysis, I2 method was also used efficiently check the heterogeneity present among the selected studies [20]. Hardy-Weinberg equilibrium (HWE) in the controls was estimated using chi-square test. The asymmetry of funnel plot was calculated using Egger's linear regression test (a type of linear regression approach) which measures the mentioned parameter on the natural logarithm scale of the ORs. T-test was employed to determine the significance of intercept wherein where p-value <0.05 represent statistically significant publication bias [21]. The entire meta-analysis mentioned in the current paper was performed by using a software named Comprehensive Meta-Analysis (CMA) V2 (Biostat, USA). The two-sided p-values were considered statistically significant when less than 0.05 for the pooled analysis.

### Trial sequential analysis (TSA):

In order to be the best, the meta-analysis has to contain all the eligible trials as per Cochrane handbook. But, it may not be able to produce sufficient satisfactory evidences. There are always chances of systematic (bias) or random (by chance) errors in meta-analysis. There, these errors were minimized using a novel statistical analysis program named Trial Sequential Analysis (TSA) tool made by Copenhagen Trial Unit, Center for Clinical Intervention Research, Denmark. It accurately estimates the required information size, adjusts threshold statistical significance and calculates the power of conclusion of the meta-analysis studies [22-24]. The crossing of TSA monitoring boundary with Z curve before reaching the required information size confirms robust evidence and further trials are considered unnecessary. The reverse condition necessitates continuous trials. Trial Sequential Analysis (version 0.9, http://www.ctu.dk/tsa/) was utilized for performing this analysis.

## Results:

### Characteristics and quality assessment of the included studies:

Table 1 and 2 (see PDF) depict the major characteristics like distribution of genotypes, minor allele frequency (MAF) in controls and cases of all the five studies included in this meta-analysis. The Figure 1 (PRISMA 2009 Flow Diagram) depicts the information required for the selection of relevant studies. These studies were assessed for their quality score as laid down by the Newcastle Ottawa scale. Almost, all of these included studies scored 5 or more stars, indicating moderate to good quality (Table 3 - see PDF).

### Diagnosis of publication bias:

The shape of the funnel plots and the results of Egger's test have negated the presence of publication bias in all the five genetic models of MnSOD Ala16Val polymorphism (Table 4; Supplementary Figure: Figure SI1) - see PDF.

### Evaluation of heterogeneity:

The Q-test and I2 statistics revealed absence of heterogeneity in Val Ala vs. Ala Ala genetic model. However, heterogeneity was observed in other four studied genetic models. Hence, random effects model was further used for synthesizing the data (Table 4 - see PDF).

### Association of MnSOD Ala16Val gene polymorphism and overall asthma risk:

**Figure 2:**
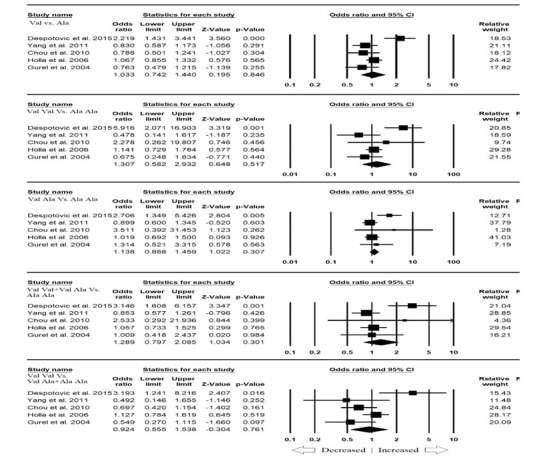
Forest plot of ORs with 95% CI of asthma risk associated with the MnSOD Ala16Val gene polymorphism for overall population. Note: Black square represents the value of OR and the size of the square indicates the inverse proportion relative to its variance. Horizontal line is the 95% CI of OR.

The pooled five pertinent and legible studies resulted into 800 confirmed asthma cases and 1009 healthy controls. All the subjects involved were scanned for the association between MnSOD Ala16Val polymorphism and overall asthma risk. The overall pooled analysis suggested no risk between MnSOD Ala16Val polymorphism and overall asthma susceptibility in all the genetic models, i.e., allelic (Val vs. Ala: p=0.846; OR=1.033, 95% CI=0.742 to 1.440), homozygous (Val Val vs. Ala Ala: p=0.517; OR=1.307, 95% CI=0.582 to 2.932), heterozygous (Val Ala vs. Ala Ala: p=0.307; OR=1.138, 95% CI=0.888 to 1.459), dominant (Val Val + Val Ala vs. Ala Ala: p=0.301; OR=1.289, 95% CI=0.797 to 2.085) and recessive (Val Val vs. Val Ala + Ala Ala: p=0.761; OR=0.924, 95% CI=0.555 to 1.538) (Figure 2).

### Association of MnSOD Ala16Val gene polymorphism and asthma risk in Asian population:

**Figure 3:**
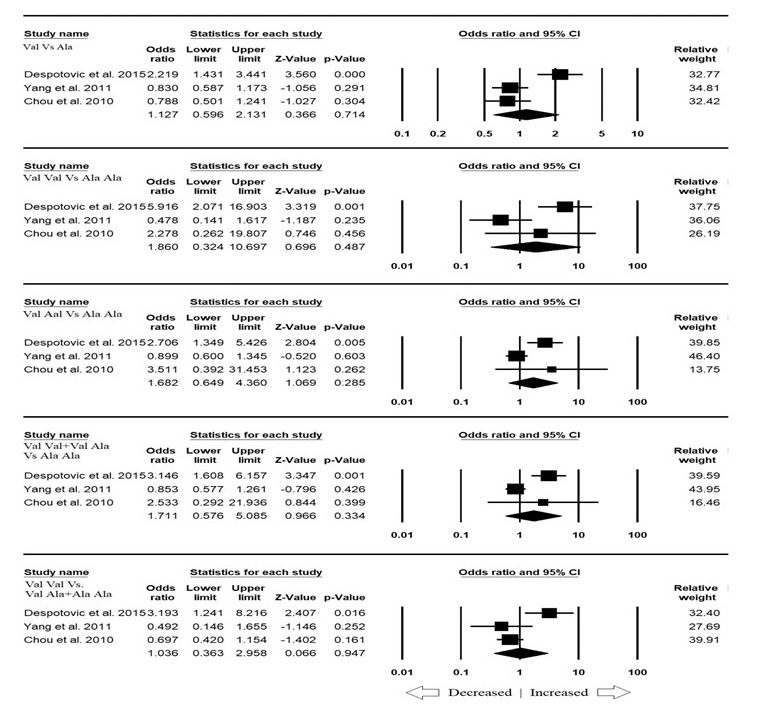
Forest plot of ORs with 95% CI of asthma risk associated with the MnSOD Ala16Val gene polymorphism for Asian subgroup population. Note: Black square represents the value of OR and the size of the square indicates the inverse proportion relative to its variance. Horizontal line is the 95% CI of OR.

The sub-group analysis of Asian ethnicity population scrutinized three studies, which involved a total number of 445 asthma cases and 577 control cases. All the studied genetic models showed heterogeneity (Table 5 - see PDF). No publication bias was found in the studies examined in this section of meta-analysis (Table 5; Figure SI2 - see PDF). On combining all the three studies, it was observed that allelic (Val vs. Ala: p=0.714; OR=1.127, 95% CI=0.596 to 2.131), homozygous (Val Val vs. Ala Ala: p=0.487; OR=1.860, 95% CI=0.324 to 10.697), heterozygous (Val Ala vs. Ala Ala: p=0.285; OR=1.682, 95% CI=0.649 to 4.360), dominant model (Val Val + Val Ala vs. Ala Ala: p=0.334; OR=1.711, 95% CI=0.576 to 5.085) and recessive (Val Val vs. Val Ala + Ala Ala: p=0.947; OR=1.036, 95% CI=0.363 to 2.958) genetic models did not reveal any association with asthma risk (Figure 3).

### Sensitivity analysis:

The leave-one-out sensitivity analysis was performed to evaluate the effect of each individual study on the overall analysis. The pooled ORs were recomputed after the exclusion of one study at a time. The sensitivity analysis did not show any differences from the primary values for overall risk (Figure SI3 - see PDF) and Asian subpopulation risk (Figure SI4 - see PDF) related to asthma. This further suggested that the results of this meta-analysis were stable and robust.

### Trial Sequential Analysis (TSA) of MnSOD Ala16Val gene polymorphism:

**Figure 4:**
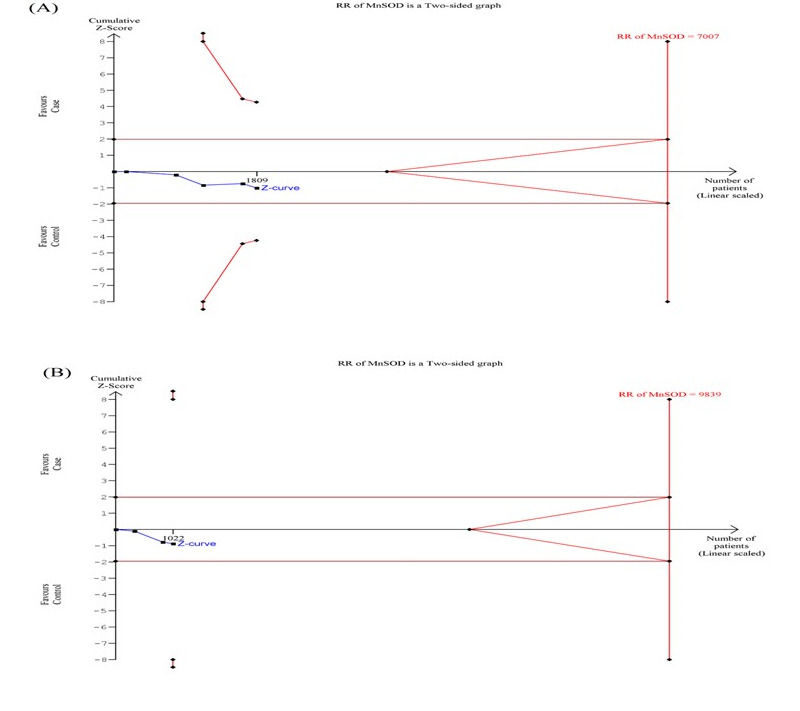
Trial sequence analysis of all the included studies dealing with MnSOD Ala16Val gene polymorphism based on dominant genetic model and asthma risk: (A) overall, (B) Asian subgroup population.

Using the data of the dominant model, the TSA analysis showed that the cumulative Z curve did not cross with TSA monitoring boundary-confirming necessity of further relevant trials for overall (Figure 4A) and Asian ethnicity (Figure 4B). TSA data for other genetic models of MnSOD Ala16Val polymorphism for overall risk and Asian population risk is shown in Figure SI5 and Figure SI6(see PDF) respectively.

## Discussion:

The presence of excess reactive oxygen species (ROS) or insufficient protection in the form of less antioxidants leads to an oxidative stress, which further contributes to the development of cutaneous disease and challenges the enzymes that metabolize ROS and other inhaled toxic compounds. ROS has been considered as vital mediators of host of cellular processes like cell adhesion, apoptosis, and the immune response [25]. The ROS also plays an important role as second messengers in intracellular signaling pathways [26]. The lungs are the major organs of the body that are regularly exposed to ROS and blood supply. Therefore, the lungs are susceptible to injury mediated by ROS [27]. As a result, the lungs have developed various endogenous antioxidant systems of their own to combat the production of free radicals [28]. The ROS produced has the capacity to alter cellular functions by affecting the activity of myriad of proteins including mitogen-activated proteins [29].

The earlier published studies have demonstrated the elevated amounts of ROS such as superoxide radicals and hydrogen peroxide (H2O2) in asthmatic patients [30]. The antioxidant enzymes control the production of ROS inside the cells. MnSOD is a major ROS detoxifying enzyme of cells because of its localization in mitochondria. This enzyme plays protective role against the damages caused by ROS and regulates the cellular concentration of O2 under both physiological and pathological conditions [31].

The genes that regulate defense mechanism in the form of antioxidants form important part of the cells' response. The cell shows this response against the production of excessive amount of ROS. The change in expression or function of MnSOD may affect mitochondrial function and the overall health of cells as a result of oxidative damage to various localized mitochondrial metabolic processes that leads to the development of different diseases. As the lungs are exposed to high oxygen concentration, changes in antioxidant defense could be one of the important mechanisms in asthma pathogenesis. The earlier published articles have many evidences showing that SNPs are a major factor in asthma susceptibility. Similar to many other common diseases, many researchers have investigated the genetics of asthma using both family-based and population-based approaches [32]. However, the results have been disappointing, therefore, the current study has tried to draw attention towards a difficult task faced by researchers to unravel the genetic architecture of asthma, a multifactorial diseases. Till date, there is no well-organized meta-analysis available that evaluated the precise association between MnSOD Ala16Val gene polymorphism and risk of asthma development. Therefore, the current meta-analysis has tried precisely to investigate this possible relationship. The current meta-analysis is based on five independent publications, which clearly produces acceptable statistical evidences that MnSOD Ala16Val variant is not a significant susceptibility factor for the asthma in overall population. Not considering the possibility that together with some other defects in the antioxidant defense system, the MnSOD Ala16Val polymorphism may become significant in inflammatory airway disease.

This gene polymorphism has a very important characteristic feature that its occurrence can vary sufficiently among different races or ethnic populations. Therefore, the current study has performed subgroup analysis of ethnicity, which further reveals no association of MnSOD Ala16Val with Asthma in the Asian population. In addition to this, the TSA analysis has further confirmed the evidences that necessary trials are needed to reach on a more precise conclusion. Earlier study revealed that the host genetic factors are potentially accountable for the development of asthma disease [33]. It has now become a widely recognized fact that the personality genetic structure is polygenic and complex in nature, wherein many genes participates in small ways to individual differences in specific personality dimensions. Therefore, a single genetic variant is generally considered as insufficient to predict the susceptibility towards this problematic disease.

Although, the current trial sequential meta-analysis has achieved various important findings, still it suffers from few limitations. First, the heterogeneity existed in between the studies used for the analysis of genetic models, which may have affected the results, although a random effects model additionally was adopted for this analysis. Second, this study has included only those studies published in the English language, which restricted the number of studies used. Third, the stratification analysis was done in sub-group ethnicity with a lesser number of individual studies that might have a small statistical power to detect the actual association, and lastly, the results are based on unadjusted estimates. The same potential confounders like age, sex, and exposure were not used to adjust the ORs in all the included studies. Notwithstanding, the above-mentioned limitations, our analysis was associated with advantages like applying NOS scale stringently did the quality assessment of the selected studies. The studies used here scored five or more stars that indicated their moderate to good quality. They had clearly mentioned the sample size, genotype, and inclusion criteria for confirmed asthma patients and healthy controls. Secondly, the publication bias was completely eliminated to give more robust conclusion. Thirdly, the likelihood of bias was minimized to the extent possible by following a thorough protocol of study identification, statistical analysis and data selection. Fourthly, sensitivity and trial sequential analysis have strengthened the conclusion of our study largely.

## Conclusion

Data shows that MnSOD Ala16Val polymorphism is not associated with asthma risk. Therefore, well-designed studies with wider spectrum of subjects from different ethnicities including the consideration of various environmental factors are warranted to comprehensively investigate the association of MnSOD Ala16Val polymorphism and asthma risk.

## Funding Statement:

This research was funded by the Scientific Research Deanship at university of Ha'il - Saudi Arabia through project number RG-I91226.

## Supplementary materials:

Supplementary file is provided as "97320630016789S1.pdf" is provided separately containing supplementary figures (Figure SI1, Figure SI2, Figure SI3, Figure SI4, Figure SI5, Figure SI6. - see PDF)
